# Digital Spatial Profiling Links Beta-2-microglobulin Expression with Immune Checkpoint Blockade Outcomes in Head and Neck Squamous Cell Carcinoma

**DOI:** 10.1158/2767-9764.CRC-22-0299

**Published:** 2023-04-11

**Authors:** Niki Gavrielatou, Ioannis Vathiotis, Thazin Nwe Aung, Saba Shafi, Sneha Burela, Aileen I. Fernandez, Myrto Moutafi, Barbara Burtness, Panagiota Economopoulou, Maria Anastasiou, Periklis Foukas, Amanda Psyrri, David L. Rimm

**Affiliations:** 1 Department of Pathology, Yale School of Medicine, New Haven, Connecticut.; 2 Department of Internal Medicine, Section of Medical Oncology, Yale School of Medicine, New Haven, Connecticut.; 3 Department of Internal Medicine, Section of Medical Oncology, Attikon University Hospital, National Kapodistrian University of Athens, Athens, Greece.

## Abstract

Programmed cell death protein‐1 (PD-1)-targeted immunotherapy is approved for recurrent or metastatic head and neck squamous cell carcinoma (R/M HNSCC) treatment. Although its efficacy correlates with PD-L1 expression, response is limited even among positive cases. We employed digital spatial profiling (DSP) to discover potential biomarkers of immunotherapy outcomes in HNSCC. Fifty prospectively collected, pretreatment biopsy samples from patients with anti-PD-1–treated R/M HNSCC, were assessed using DSP, for 71 proteins in four molecularly defined compartments (tumor, leukocyte, macrophage, and stroma). Markers were evaluated for associations with progression-free (PFS) and overall survival (OS). High beta-2 microglobulin (B2M), LAG-3, CD25, and 4-1BB in tumor; high B2M, CD45, CD4 in stroma, and low fibronectin in the macrophage compartment, correlated with prolonged PFS. Improved PFS and OS were observed for cases with high B2M by quantitative and mRNA. Findings were validated in an independent cohort for PFS (HR, 0.41; 95% confidence interval, 0.19–0.93; *P* = 0.034). B2M-high tumors showed enrichment with immune cell and immune checkpoint markers. Our study illustrates B2M expression is associated with improved survival for immune checkpoint inhibitor (ICI)-treated HNSCC.

**Significance:**

In the current study, DSP revealed the positive association of B2M expression in the tumor compartment with immunotherapy outcomes in R/M HNSCC.

## Introduction

Immune checkpoint blockade with the mAb pembrolizumab, targeting programmed death-1 (PD-1), is the standard of care (SOC) in first-line management of recurrent or metastatic head and neck squamous cell carcinoma (R/M HNSCC), while both pembrolizumab and the PD-1–directed antibody nivolumab are standard for platinum-refractory HNSCC (1–4). The efficacy of pembrolizumab has been shown to directly correlate with the levels of expression of the programmed death-ligand 1 (PD-L1) protein (1, 2, 5, 6). However, pembrolizumab alone or combined with chemotherapy has shown no improvement for progression-free survival (PFS) compared with SOC, and objective response rate is less than 20%, even for PD-L1–positive cases, indicating the need to find biomarkers that will enable optimized patient selection, as well as to identify and overcome the underlying mechanisms of immunotherapy resistance.

Incorporation of immunotherapy agents in cancer treatment paradigms restores function to host antitumor immunity, controlling disease progression and optimally leading to objective tumor response. Consequently, HNSCC immunotherapy biomarker studies have been primarily focused on the dynamic interactions between tumor cells and immune cells residing in the tumor microenvironment (TME), both at protein and molecular level (7).Here, we employed the GeoMx digital spatial profiling (DSP) platform to investigate the effect of 71 candidate proteins, measured in four molecularly defined tissue compartments on immunotherapy outcomes in patients with R/M HNSCC. Results were validated both orthogonally using quantitative immunofluorescence (QIF) and spatial RNA *in situ* on our discovery cohort, and externally, by QIF on an independent, validation cohort of R/M HNSCC cases.

## Materials and Methods

### Discovery and Validation Cohorts

Our discovery cohort (Athens cohort, YTMA496) consisted of prospectively collected pretreatment biopsy samples from 50 patients with R/M HNSCC, enrolled in the NCT#03652142 study and treated with nivolumab at Attikon University Hospital, National Kapodistrian University of Athens, from 2017 to 2020. Our validation cohort (Yale cohort, YTMA523) comprised retrospectively collected pretreatment biopsy samples from 29 R/M HNSCC cases, treated with ICI at Yale New Haven Hospital from 2014 to 2020.

Pathologist selected, representative tumor areas were included in two independent tissue microarray (TMA) paraffin blocks for each cohort, each containing one nonadjacent 0.6 mm tumor-tissue core per case. Two slides of 5-μm-thick tissue sections, each obtained from a different TMA block, were used for all assays. Immunotherapy outcomes were collected for all cases, including assessment of best overall response (BOR), defined by RECIST version 1.1., as complete response (CR), partial response (PR), stable disease (SD), and progressive disease, and cases were annotated for response (CR, PR) and disease control (CR, PR, SD). PFS and overall survival (OS) following immunotherapy initiation were also determined. Clinical and epidemiologic characteristics of both cohorts are summarized in Supplementary Table S1. Written informed consent or waiver of consent was provided by all participating patients. The NCT#03652142 study protocol was approved by the Ethics Committee/Institutional Review Board of Attikon University Hospital, Haidari, Athens, Greece (protocol # ΒΠΠΚ, ΕΒΔ2840/21-11-2017), the current study was approved by the Yale Human Investigation Committee protocol #9505008219 and both were conducted in accordance with the Declaration of Helsinki.

### DSP

**FIGURE 1:**
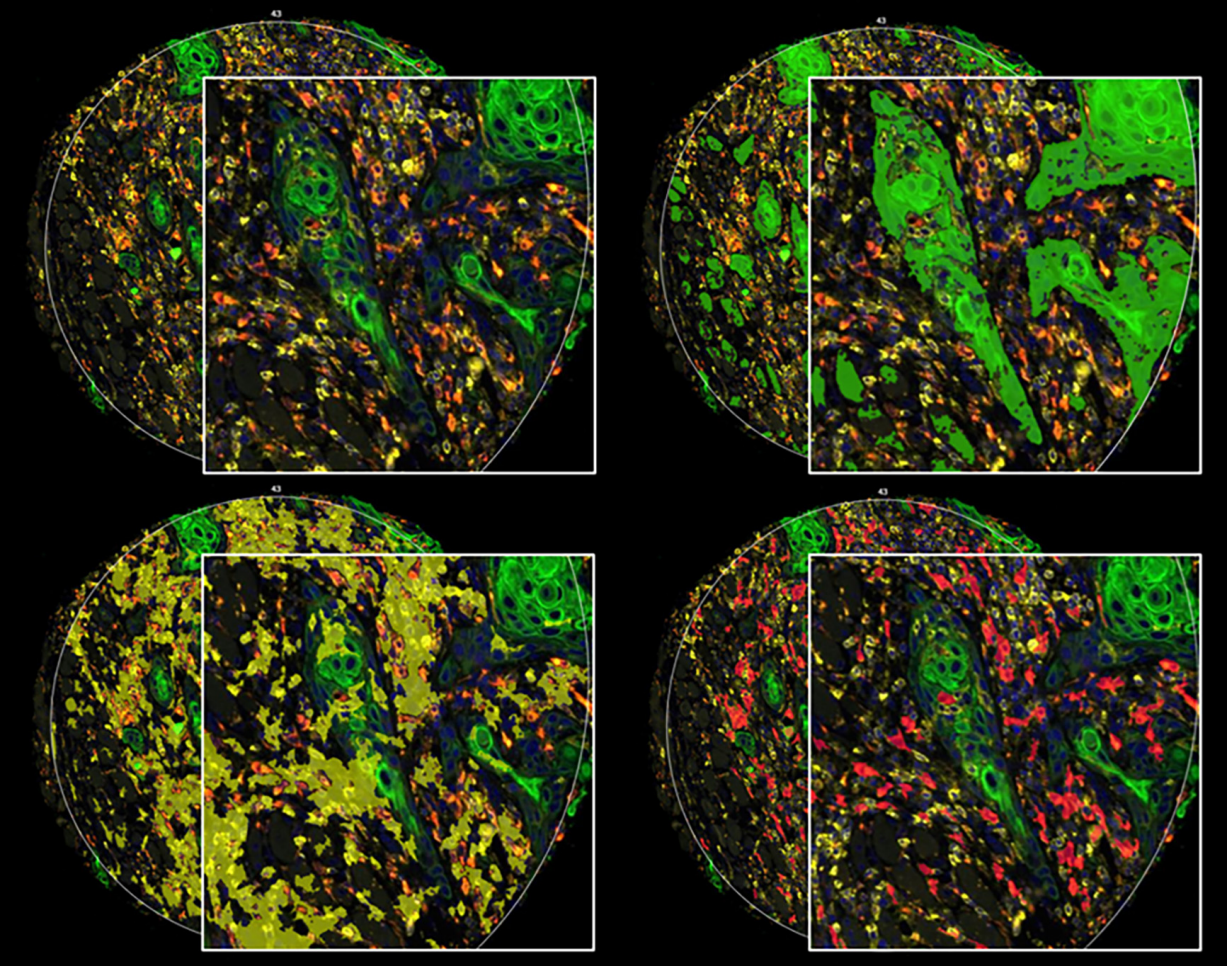
Molecular tissue compartmentalization of a representative tumor core (YTMA496, block1), using the GeoMx DSP instrument. Visualization of PanCK, CD45, and CD68 signal localization was used to characterize the tumor (green), leukocyte (yellow), and macrophage (red) compartment, respectively.

Following deparaffinization and antigen retrieval, YTMA496 slides were processed with overnight incubation with three fluorescently-labeled antibodies, for the cellular characterization of three distinct tissue compartments: tumor (pan-cytokeratin PanCK+), leukocyte (CD45^+^), and macrophage (CD68^+^), as well as with a previously vendor-validated, 77-oligonucleotide–conjugated primary antibody cocktail, for the simultaneous detection of 71 target proteins and six control markers (Supplementary Table S2). Next, after nuclear staining and tissue fixation, slides were loaded in the GeoMx digital spatial profiler for scanning and annotation of regions of interest (ROI), covering a maximum diameter of 600 μm for each TMA core. Each ROI was then further divided into tumor, leukocyte, and macrophage compartments based on the spatial distribution of the fluorescence signal from the PanCK, CD45, and CD68 antibodies, respectively (Fig. 1). Then, via an automated procedure, sequential exposure of each compartment to UV light prompted the photocleavage of oligonucleotides from their specific antibodies, followed by their aspiration and collection into a 96-well plate. Upon hybridization with four-color, six-spot optical barcodes, oligonucleotides were counted and “translated” into expression levels of their corresponding proteins, using the nCounter platform (NanoString Technologies). Digital raw counts were normalized with internal spike-in controls, followed by quality control and additional normalization, separately for each compartment, by the geometric mean of two housekeeping isotype controls (Histone H3, S6). Background ratio (signal to noise) was defined for each compartment using the geometric mean of the three negative isotype controls (Ms IgG1, Ms IgG2a, and Rb IgG; Supplementary Fig. S1). Finally, to produce the stromal compartment, counts from both the leukocyte and macrophage compartments were normalized together and their summation for each marker represented the stromal protein expression of each ROI.

### Multiplexed Immunofluorescence Staining

To orthogonally validate our findings using DSP technology, we performed multiplexed (5-plex) QIF with a staining protocol for PanCK/CD45/CD68 and beta2-microglobulin (B2M) on tissue sections from two independent blocks from both the Athens cohort (YTMA496) and the Yale cohort (YTMA523). Protocol details can be found in Supplementary Materials and Methods.

### RNA-ISH Analysis

Available mRNA *in situ* detection using DSP technology from YTMA496 was utilized as an additional orthogonal validation step. Future work will describe these data in more detail, but here mRNA *in situ* data were only used to assess the effect of *B2M* expression in the tumor compartment, with the aim to further support our protein findings. Briefly, high-plex spatial detection of mRNA *in situ* was performed according to the NanoString GeoMX RNA assay protocol (MAN-10087-03) using the Whole Transcriptome Atlas probe reagent, as has been described previously (8), for the same three morphology marker–defined compartments that were investigated in the DSP protein assay; tumor (PanCK), leukocyte (CD45), and macrophage (CD68). Library preparation was also performed according to manufacturer's protocol and three pooled libraries were created—one for each compartment. Following library quality assessment, samples were sequenced using the Illumina NovaSeq platform. Raw sequencing reads were obtained after combining the Illumina sequencing FASTQ files and the NanoString GeoMx DSP configuration files, and digital count conversion files were created and uploaded into the GeoMx DSP Instrument. Finally, quality control and data processing was performed, followed by Q3 normalization (i.e., the upper third quartile of all selected targets was used for the normalization of all targets above the limit of quantitation).

### The Cancer Genome Atlas HNSCC Cohort Analysis

To evaluate a potential prognostic implication of *B2M* gene expression in HNSCC, we used publicly available data from 521 patients included in The Cancer Genome Atlas (TCGA) HNSCC cohort (9, 10) and investigated the association of *B2M* with OS.

### Statistical Analysis

For the DSP data analysis, the average of digital protein counts acquired from each block of YTMA496 was calculated in each tissue compartment, for all cases. Patients were dichotomized to either high or low expressors using the median and upper tertile as exploratory cutoffs for each marker. A univariate Cox regression model was used for PFS and OS analysis of all quantified protein markers. Average QIF scores were also obtained from matched patient cores of the YTMA496 and YTMA523 blocks and Spearman rank correlation coefficient (*R*) was used to test the linear association between DSP counts and QIF scores of B2M in the tumor compartment. Cases were divided into high and low groups by comparing the upper tertile with the two lower tertiles of tumor B2M expression. Multivariate analysis was also performed, both for PFS and OS, using a Cox proportional hazards model of established prognostic clinical variables. For the YTMA496 RNA *in situ* data analysis, counts of *B2M* expression in the tumor compartment were also explored for associations with PFS and OS, after stratification of patients to high and low expressors by tertiles. The Mann–Whitney *U* test was used for the comparison of immune cell and immune checkpoint levels of expression between “B2M-high” and “B2M-low” tumors, again stratified by the upper tertile. The prognostic significance of *B2M* expression in TCGA HNSCC cohort was evaluated using the “Gene_Outcome Module” of TIMER 2.0 platform and the upper tertile cutoff (11). All hypothesis testings were performed at a two-sided level of significance (*P* = 0.05). Statistical analyses were performed using GraphPad Prism v9.3.0 software (GraphPad Software, RRID:SCR_002798) and R studio v1.4.1717.

### Data Availability

The mRNA *in situ* data generated in this study have been deposited in NCBI's Gene Expression Omnibus (GEO) and are accessible through GEO Series accession number GSE226134. TCGA-HNSC data can be accessed at https://cancergenome.nih.gov. The protein expression data have been deposited in Yale AQUAmine repository and are available upon request from the corresponding author.

## Results

For the initial search for relationships between DSP compartment specific expression and outcome, we performed univariate analysis. The DSP protein data from our discovery cohort, identified in four different molecularly defined compartments (tumor, leukocyte, macrophage, and stroma), revealed the association of eight markers with either PFS or OS. This included three proteins involved in antigen presentation: B2M, a MHC class I component, as well as dendritic cell markers CD11c and CD25 (Table 1). Patients in the upper tertile of B2M expression in tumor, were found to have significantly prolonged PFS and OS (*P*_unadjusted_ = 0.010 and *P* = 0.021, respectively) to immunotherapy.

**TABLE 1:** Protein markers significantly associated with PFS and OS of patients with immunotherapy-treated R/M HNSCC (Athens cohort). Discovery set univariate analysis of DSP results

Outcome	Compartment	Marker	Cutoff	Log-rank *P* value (unadjusted)	Univariate HR (95% CI)
PFS	Tumor	B2M	Upper tertile	0.010	0.36 (0.17–0.75)
		LAG-3	Upper tertile	0.029	0.42 (0.20–0.89)
		CD25	Upper tertile	0.017	0.38 (0.18–0.85)
		4-1BB	Median	0.030	0.46 (0.21–0.98)
	Stroma	CD45	Median	0.002	0.32 (0.14–0.74)
		CD4	Median	0.004	0.35 (0.15, 0.80)
		B2M	Median	0.026	0.43 (0.19, 0.89)
	Macrophage	Fibronectin	Median	0.027	2.20 (1.02, 4.75)
OS	Tumor	B2M	Upper tertile	0.021	0.33 (0.14–0.74)
		CD25	Upper tertile	0.023	0.33 (0.15–0.75)
	Stroma	CD11c	Median	0.023	0.39 (0.16–0.92)

Abbreviations: CI, confidence interval; HR, hazard ratio; OS, overall survival; PFS, progression free survival.

**FIGURE 2:**
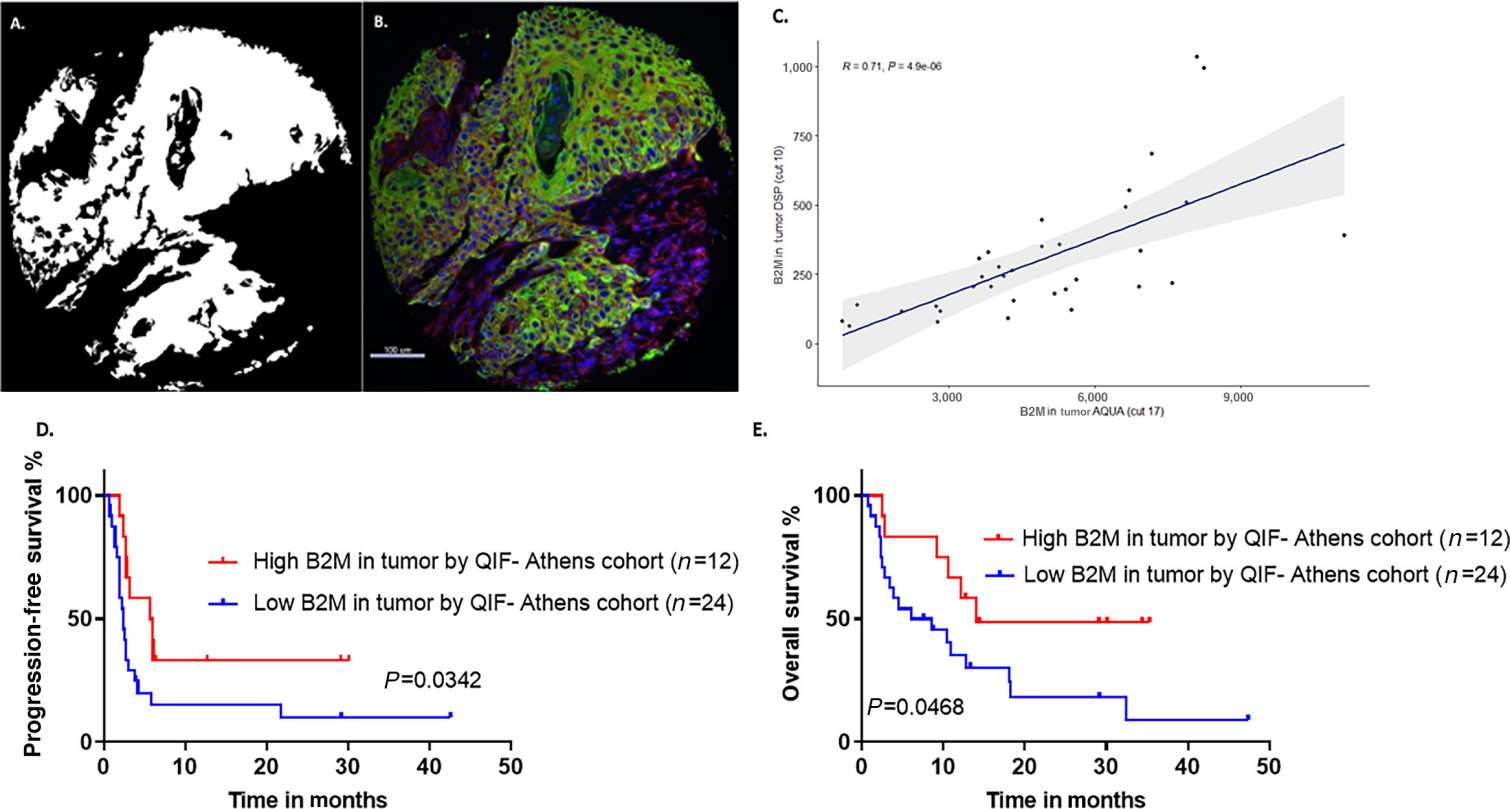
**A** and **B,** Representative images of B2M immunofluorescence staining (Athens cohort, YTMA496). Tumor mask (white) and pseudocolor image depicting B2M (red), CK (green), and DAPI (blue) signal. **C,** Correlation of DSP and QIF scores of B2M expression in tumor. PFS (**D**) and OS (**E**) curves comparing the effect of high versus low B2M expression in tumor by QIF. B2M, beta-2 microglobulin; PFS, progression-free survival; OS, overall survival; QIF, quantitative immunofluorescence.

Next, we sought to validate the association of B2M with PFS and OS using an orthogonal method of QIF (AQUA) on our discovery cohort (Fig. 2A and B; Supplementary Fig. S2A). First, DSP B2M counts in tumor were correlated with the corresponding QIF B2M scores for the same cases and high concordance was observed between the two assays (*R* = 0.71, *P* = 4.9e-06; Fig. 2C). In addition, cases at the top tertile of B2M expression in tumor by QIF were associated with improved PFS and OS [HR, 0.43; 95% confidence interval (CI), 0.21–0.9; *P* = 0.034 and HR, 0.41; 95% CI, 0.18–0.90; *P* = 0.047, respectively] (Fig. 2D and E). This association was maintained in multivariate analysis (Supplementary Fig. S2B and S2C). Notably, no significant association was observed with respect to BOR or disease control (Supplementary Fig. S2D and S2E), potentially due to the small sample size, because only 7 responders and 13 patients with disease control were included in the analysis.

**FIGURE 3:**
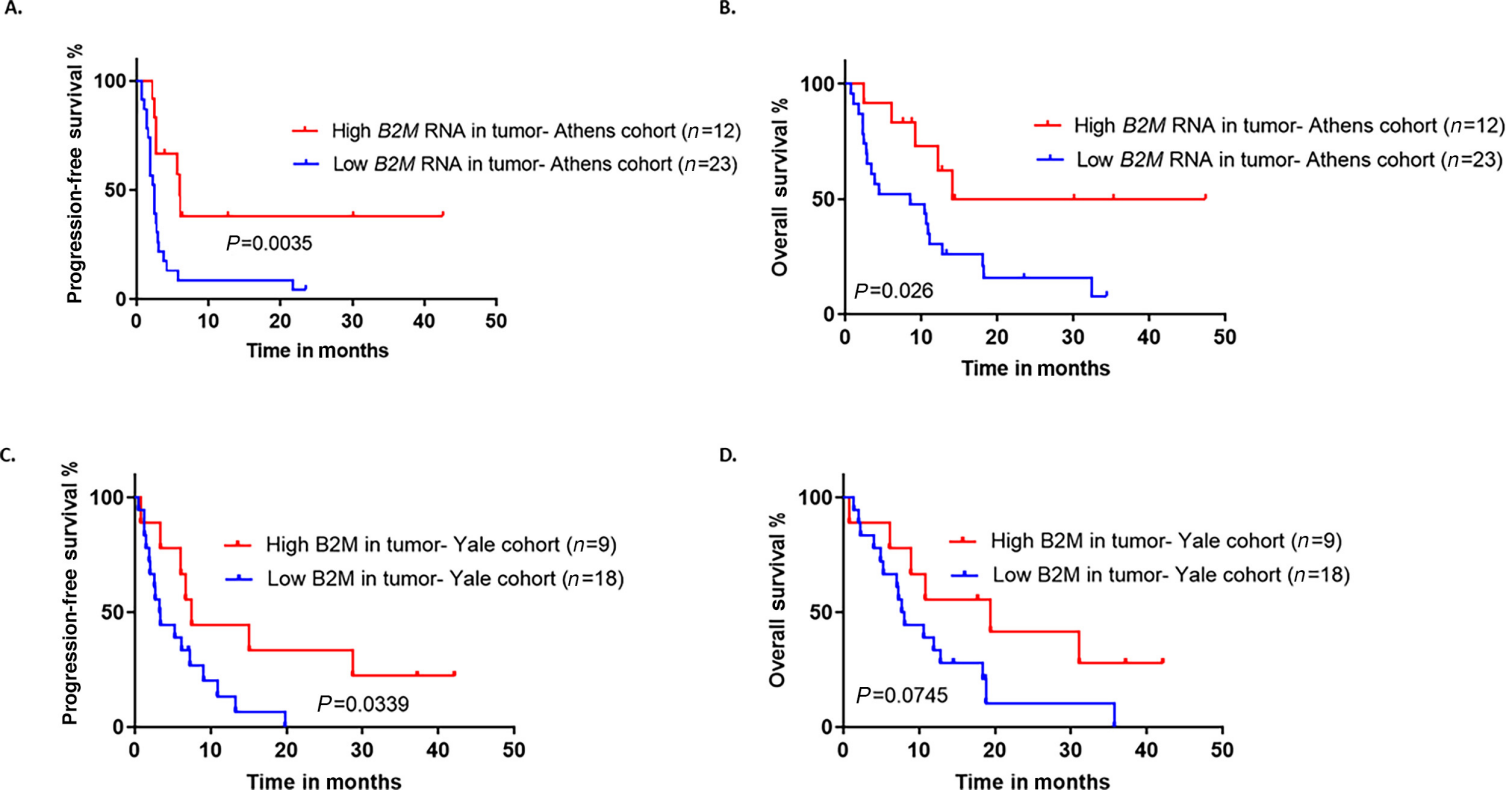
**A** and **B,** High B2M gene expression is associated with prolonged PFS and OS in Athens cohort (YTMA496). Similarly, in the independent, validation, Yale cohort (YTMA523), high B2M levels by QIF, were associated with improved PFS (**C**) and showed the same trend for OS (**D**). B2M, beta-2 microglobulin; PFS, progression-free survival; OS, overall survival; QIF, quantitative immunofluorescence.

While mRNA and protein are not always correlated, we used spatially characterized RNA *in situ* data from the same cohort (Athens, YTMA496) to investigate the effect of *B2M* gene expression on survival, as a second orthogonal validation step. B2M protein expression in tumor showed a strong agreement with the RNA levels of *B2M* expression (Supplementary Fig. S3A). High *B2M* expression, defined by the same cutoff (upper tertile), was also associated with prolonged PFS [HR, 0.32; 95% CI, 0.15–0.65; *P* = 0.0035] and OS [HR, 0.35; 95% CI, 0.16–0.77; *P* = 0.026] (Fig. 3A and B). Similar to protein findings, no association was observed between *B2M* levels and BOR or disease control (Supplementary Fig. S3B and S3C).

Next, B2M expression in tumor was quantified and explored for associations with survival, by QIF on a second, independent, validation cohort of R/M HNSCC cases (Yale cohort, YTMA523; Supplementary Fig. S4A). In concordance with our previous observations, high B2M expression in the tumor compartment showed significant association with increased PFS [HR, 0.41; 95% CI, 0.19–0.93; *P* = 0.034] and had a similar trend for OS [HR, 0.44; 95% CI, 0.19–1.0; *P* = 0.074] (Fig. 3C and D) and maintained the same effect in multivariate analysis (Supplementary Fig. S4B and S4C). Tumor B2M expression was also not associated with response or disease control for patients in the Yale cohort (Supplementary Fig. S4D and S4E).

*B2M* expression was not associated with OS in the non–immunotherapy-treated TCGA HNSCC cohort [HR, 1.071; 95% CI, 0.924–1.242; *P* = 0.363] (Supplementary Fig. S5).

Finally, in an effort to approach the underlying mechanisms of the observed synergy of B2M expression with ICI efficacy, we hypothesized the presence of increased immunogenicity among B2M-high tumors, and thus, we investigated the differential expression of immune cell and immune checkpoint markers between B2M-high and B2M-low cases. B2M-high tumors demonstrated significantly increased immune cell infiltration and immune checkpoint expression, according to DSP data (Supplementary Fig. S6).

## Discussion

The therapeutic effect of PD-1/PD-L1 axis inhibition is thought to be primarily based on the reactivation of previously exhausted T cells and the incitement of antitumor response via increased cytotoxicity (12). Successful adaptive antitumor immunity has been described to be closely tied to host recognition of cancer cell–specific proteins, displayed on the surface of tumor cells as peptide–MHC-I complexes, called cancer neoantigens (13). B2M is a critical component of the MHC-I structure, thus B2M expression represents a surrogate metric of MHC-I integrity. B2M loss or mutation, and consequently deficient MHC-I antigen presentation, is a well-described mechanism of tumor-intrinsic resistance for immunotherapy-treated patients in several melanoma studies (14, 15). Similarly, in non–small cell lung cancer, progressive, *de novo*, abrogation of MHC-I expression, has been described as a means of acquired resistance to immunotherapy (16). In head and neck cancer, reports on the prognostic significance of B2M have been contradictory; high B2M levels have been associated with poor survival and increased metastatic potential in patients treated with primary surgery (17), while other reports suggest a negative prognostic impact of MHC-I loss, due to restricted immunosurveillance (18, 19).

While MHC-I loss or downregulation has been proposed as a mechanism of tumor immune evasion in HNSCC, its direct effect on anti-PD-1 treatment outcomes has yet to be elucidated. Here, we found an association of pretreatment B2M expression with PFS in patients with R/M HNSCC treated with ICI. A statistically significant association with BOR or disease control was not found, presumably due to the small number of responders/patients demonstrating disease control. It has been suggested that intermediate MHC-I downregulation might be overcome by immunotherapy, while severe MHC-I downregulation is required to evoke treatment resistance (20). Conversely, our findings indicate that survival benefit is seen when the antigen-presenting machinery is highly expressed, as was represented by the subgroup at the highest tertile of B2M expression, potentially compensating for the highly immunosuppressive nature of HNSCC TME. In addition, we observed an increased infiltration by immune cells, as well as higher immune checkpoint expression among B2M-high cases, predominantly in the tumor compartment. This is consistent with the notion that “inflamed” tumors are associated with intact proximal steps of the Cancer-Immunity Cycle and may not require further enhancement (21).

There are several limitations to this study. First, all assays were performed on TMA biopsy samples rather than whole tissue sections. Higher accuracy in sample representation was pursued by preserving 2-fold redundancy in all assays (two tumor cores from each case were assessed); however, tissue heterogeneity might not have been adequately portrayed. A second limitation of this work is the validation cohort. In contrast to the prospectively collected discovery cohort, our validation cohort was retrospectively collected and included fewer cases. However, this cohort is essentially serially collected and thus representative of R/M HNSCC incidence in Yale New Haven Hospital. As a result, our findings need to be further investigated in larger, prospectively collected cohorts.

In conclusion, among several other markers assessed in multiple tissue compartments, B2M expression in tumor cells exhibited a strong association with immunotherapy outcomes in R/M HNSCC, an effect that was reproduced in an additional, validation cohort. Our results propose the further validation toward implementation of this broadly applicable, yet currently overlooked, biomarker. We propose that, once validated, it could have value in the clinical setting as an initial screening method, aiming at the optimization of HNSCC treatment paradigms.

## Supplementary Material

Supplemental Figure 1 signal to noise ratio plots for tumor, leukocyte and macrophage compartments

Supplemental Figure 2 b2m expression range, multivariate analysis of pfs and os and association with response and disease control in Athens cohort

Supplemental Figure 3 correlation of b2m protein with B2M mRNA expression in Athens cohort. Association of B2M mRNA levels with response and disease control

Supplemental Figure 4 b2m expression range, multivariate analysis of pfs and os and association with response and disease control in Yale cohort

Supplemental Figure 5 association of B2M mRNA expression with OS in the non-immunotherapy treated TCGA HNSCC ccohort

Supplemental Figure 6 expression of immune-related and immune-checkpoint markers in B2M high vs low groups

Supplemental Table 1 clinical characteristics of discovery (Athens) and validation (Yale) cohorts

Supplemental Table 2 Target-protein panels included in the GeoMxTM Immuno-Oncology Human Protein Assay
