# Supplementary material for: Digital Spatial Profiling Links Beta-2-microglobulin Expression with Immune Checkpoint Blockade Outcomes in Head and Neck Squamous Cell Carcinoma
Source: Cancer Res Commun. 2023 Apr 11;3(4):558–63. doi: 10.1158/2767-9764.CRC-22-0299 (PMC10088911; doi:10.1158/2767-9764.CRC-22-0299)
Supplement: Supplemental Figure 1 — signal to noise ratio plots for tumor, leukocyte and macrophage compartments [file crc-22-0299-s01.pdf]

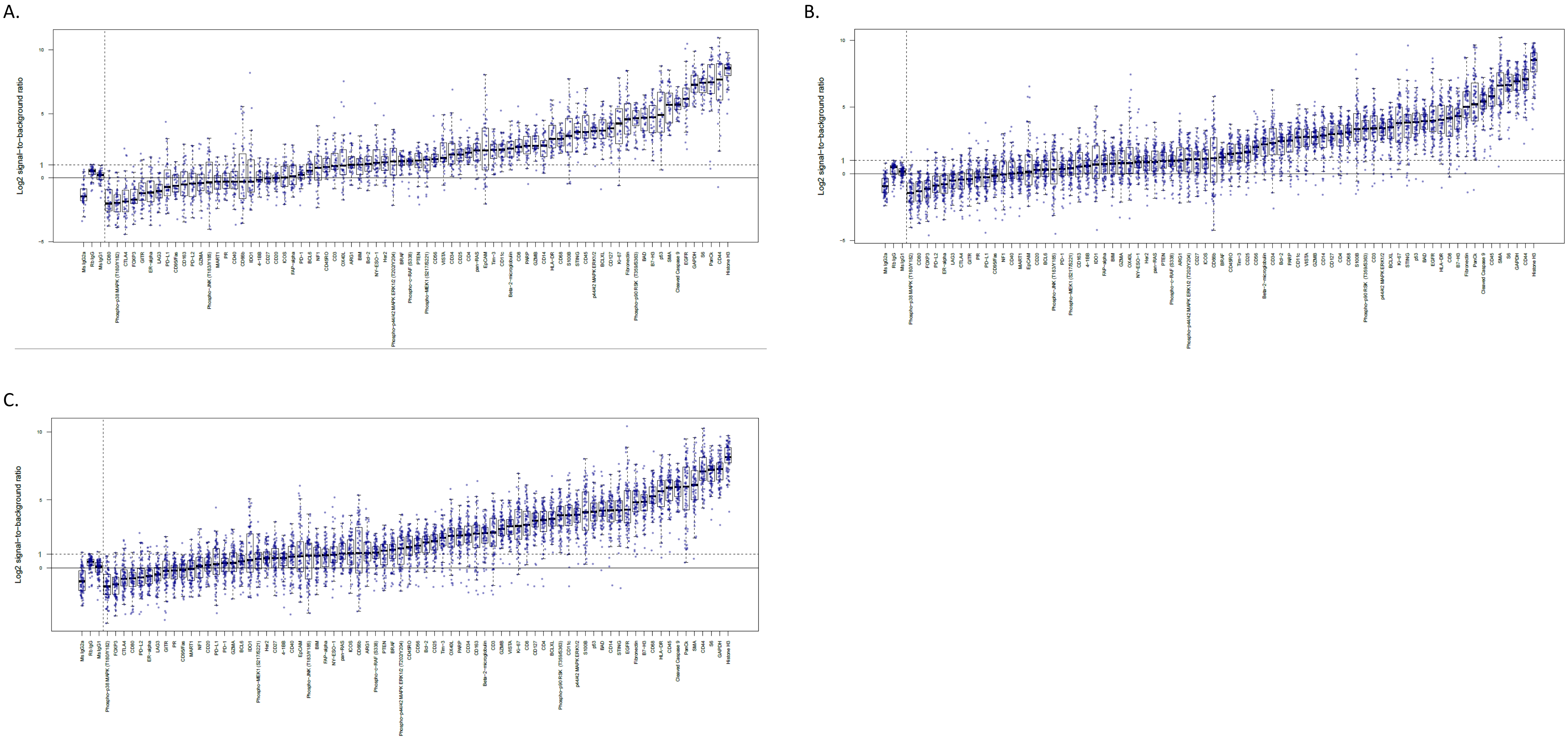

Supplemental Figure 1. Signal to noise ratio plot depicting the level of expression of all measured proteins in each ROI in the **A**. tumor **B.** leukocyte and **C.** macrophage compartment.
