# Supplementary material for: Digital Spatial Profiling Links Beta-2-microglobulin Expression with Immune Checkpoint Blockade Outcomes in Head and Neck Squamous Cell Carcinoma
Source: Cancer Res Commun. 2023 Apr 11;3(4):558–63. doi: 10.1158/2767-9764.CRC-22-0299 (PMC10088911; doi:10.1158/2767-9764.CRC-22-0299)
Supplement: Supplemental Figure 2 — b2m expression range, multivariate analysis of pfs and os and association with response and disease control in Athens cohort [file crc-22-0299-s02.pdf]

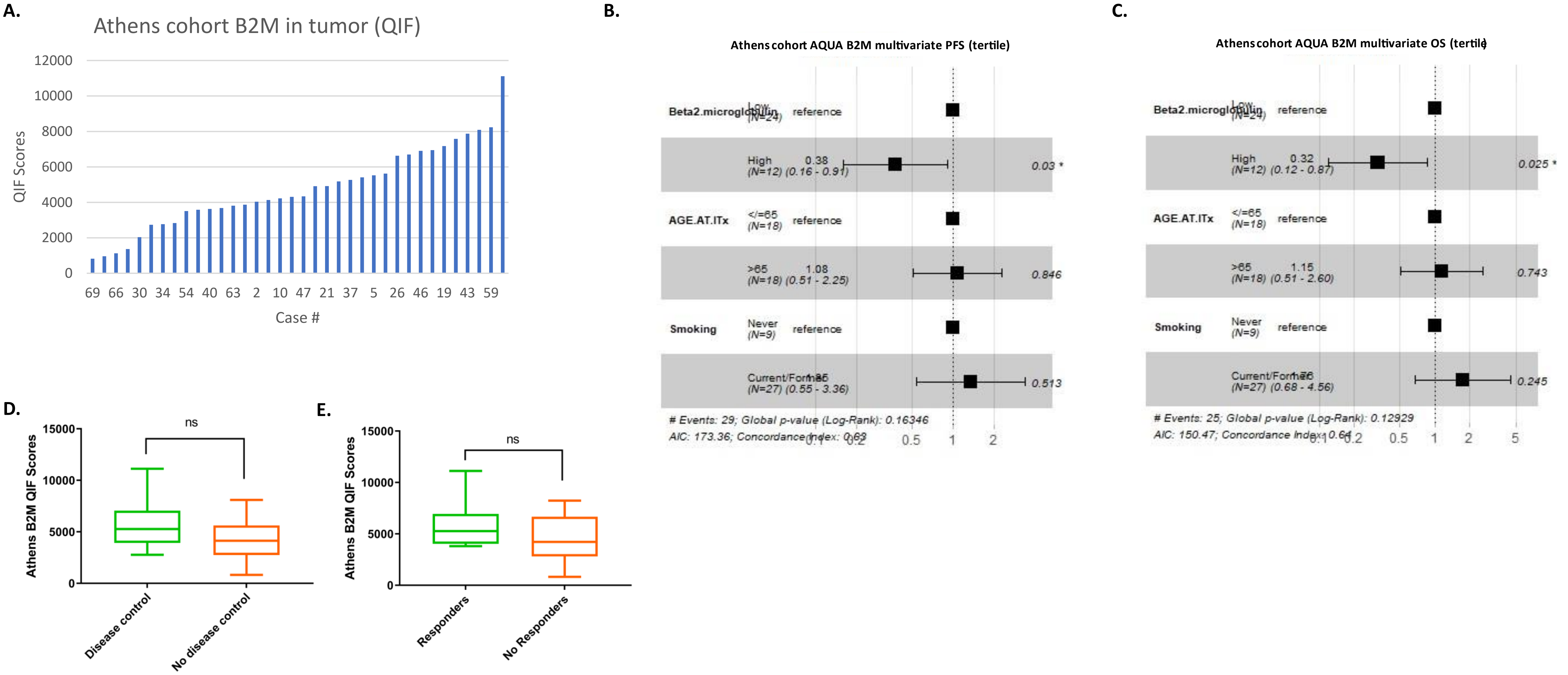

Supplemental Figure 2. Athens cohort **A**. Dynamic range of B2M expression in tumor by QIF. **B,C.** Tumor B2M expression remained significant for PFS and OS, in multivariate analysis, after adjusting for patient age and smoking status. B2M expression in tumor was not significantly associated with response **D.** or disease control **E.**
