# Supplementary material for: Digital Spatial Profiling Links Beta-2-microglobulin Expression with Immune Checkpoint Blockade Outcomes in Head and Neck Squamous Cell Carcinoma
Source: Cancer Res Commun. 2023 Apr 11;3(4):558–63. doi: 10.1158/2767-9764.CRC-22-0299 (PMC10088911; doi:10.1158/2767-9764.CRC-22-0299)
Supplement: Supplemental Figure 3 — correlation of b2m protein with B2M mRNA expression in Athens cohort. Association of B2M mRNA levels with response and disease control [file crc-22-0299-s03.pdf]

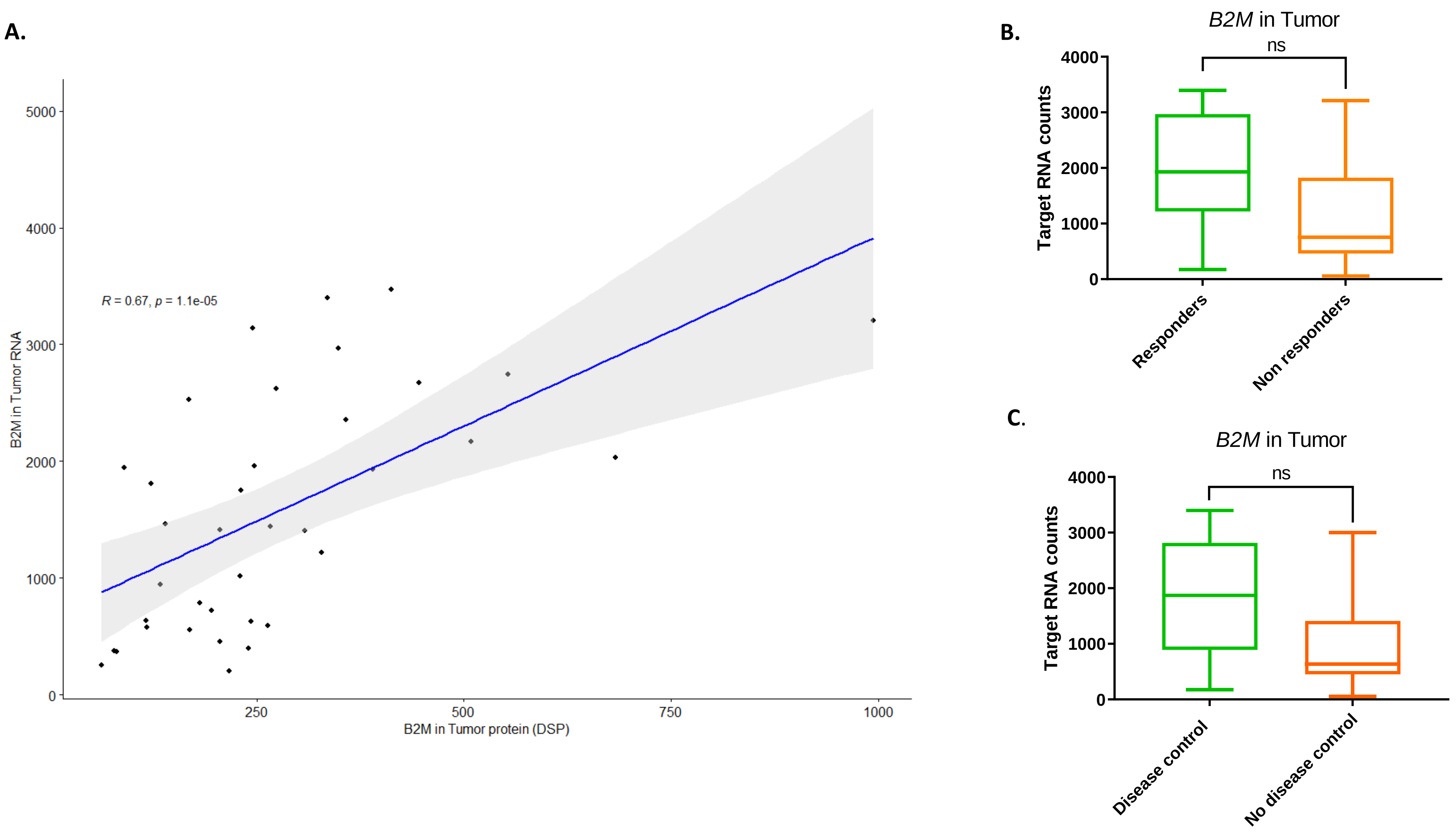

Supplemental Figure 3. A. Correlation of B2M protein levels with *B2M* mRNA expression in Athens cohort. *B2M* expression showed no association with response **B.** or disease control **C.**
