# Supplementary material for: Digital Spatial Profiling Links Beta-2-microglobulin Expression with Immune Checkpoint Blockade Outcomes in Head and Neck Squamous Cell Carcinoma
Source: Cancer Res Commun. 2023 Apr 11;3(4):558–63. doi: 10.1158/2767-9764.CRC-22-0299 (PMC10088911; doi:10.1158/2767-9764.CRC-22-0299)
Supplement: Supplemental Figure 4 — b2m expression range, multivariate analysis of pfs and os and association with response and disease control in Yale cohort [file crc-22-0299-s04.pdf]

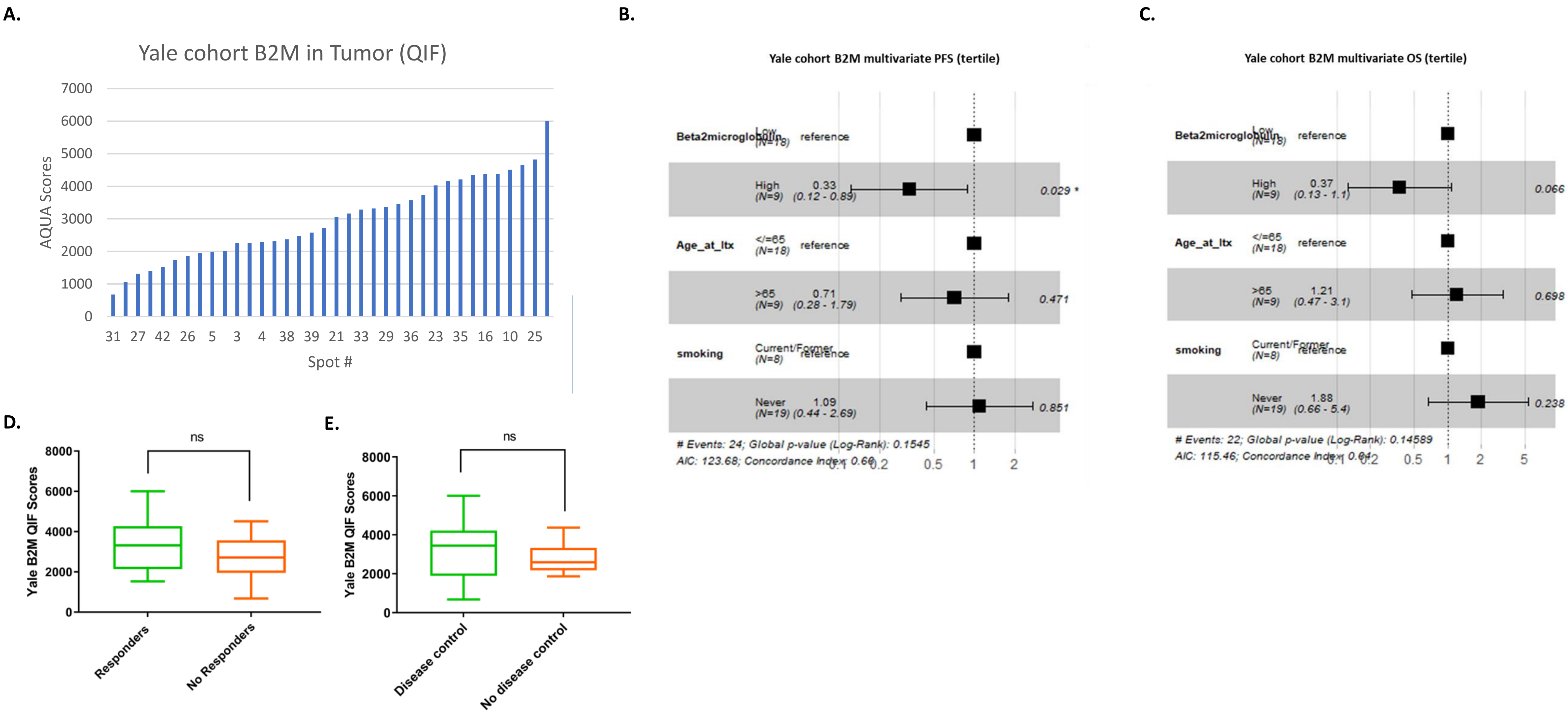

Supplemental Figure 4. Yale cohort **A**. Dynamic range of B2M expression in tumor by QIF. **B,C.** Tumor B2M expression remained significant for PFS and maintained the same trend for OS, in multivariate analysis, after adjusting for patient age and smoking status. B2M expression in tumor was not significantly associated with response **D.** or disease control **E.**
