# Supplementary material for: Digital Spatial Profiling Links Beta-2-microglobulin Expression with Immune Checkpoint Blockade Outcomes in Head and Neck Squamous Cell Carcinoma
Source: Cancer Res Commun. 2023 Apr 11;3(4):558–63. doi: 10.1158/2767-9764.CRC-22-0299 (PMC10088911; doi:10.1158/2767-9764.CRC-22-0299)
Supplement: Supplemental Figure 5 — association of B2M mRNA expression with OS in the non-immunotherapy treated TCGA HNSCC ccohort [file crc-22-0299-s05.pdf]

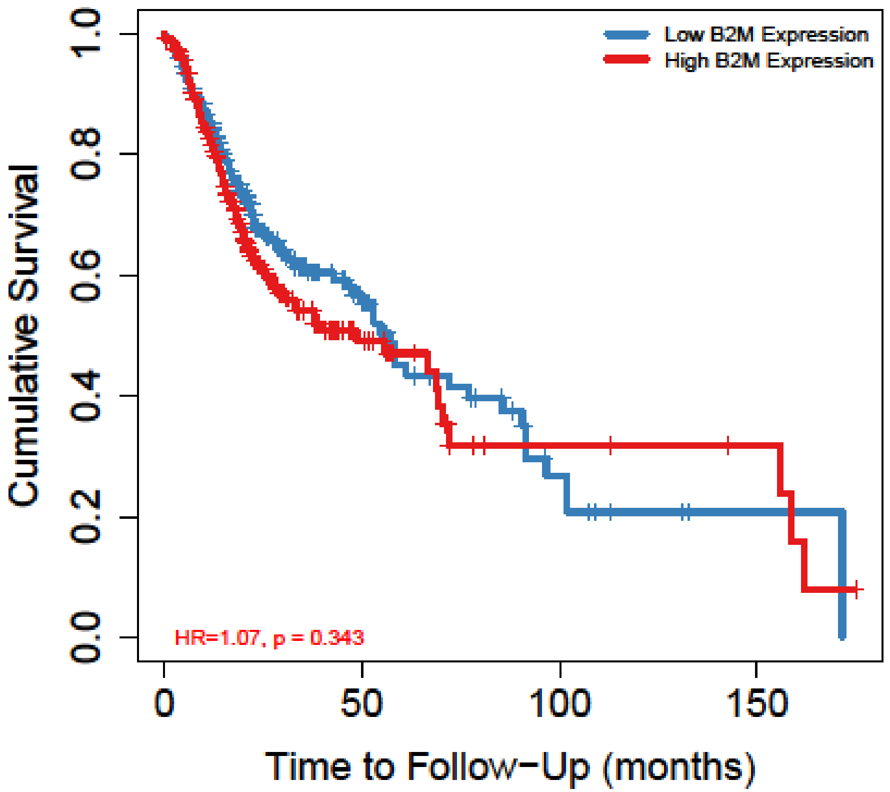


```
B2M in HNSC (n=522):
Model: Surv(OS, EVENT) ~ `B2M`
521 patients with 222 dying ( 1 missing obs. )
     coef    HR se(coef) 95%CI_l 95%CI_u      z      p signif
B2M 0.069 1.071    0.076   0.924   1.242 0.909 0.363
Rsquare = 0.002 (max possible = 9.91e-01 )
Likelihood ratio test p = 3.59e-01
Wald test p = 3.63e-01
Score (logrank) test p = 3.63e-01
```

Supplemental Figure 5. *B2M* expression is not associated with overall survival in the TCGA-HNSCC cohort (graph exported from TIMER 2.0).
