# Supplementary material for: Digital Spatial Profiling Links Beta-2-microglobulin Expression with Immune Checkpoint Blockade Outcomes in Head and Neck Squamous Cell Carcinoma
Source: Cancer Res Commun. 2023 Apr 11;3(4):558–63. doi: 10.1158/2767-9764.CRC-22-0299 (PMC10088911; doi:10.1158/2767-9764.CRC-22-0299)
Supplement: Supplemental Figure 6 — expression of immune-related and immune-checkpoint markers in B2M high vs low groups [file crc-22-0299-s06.pdf]

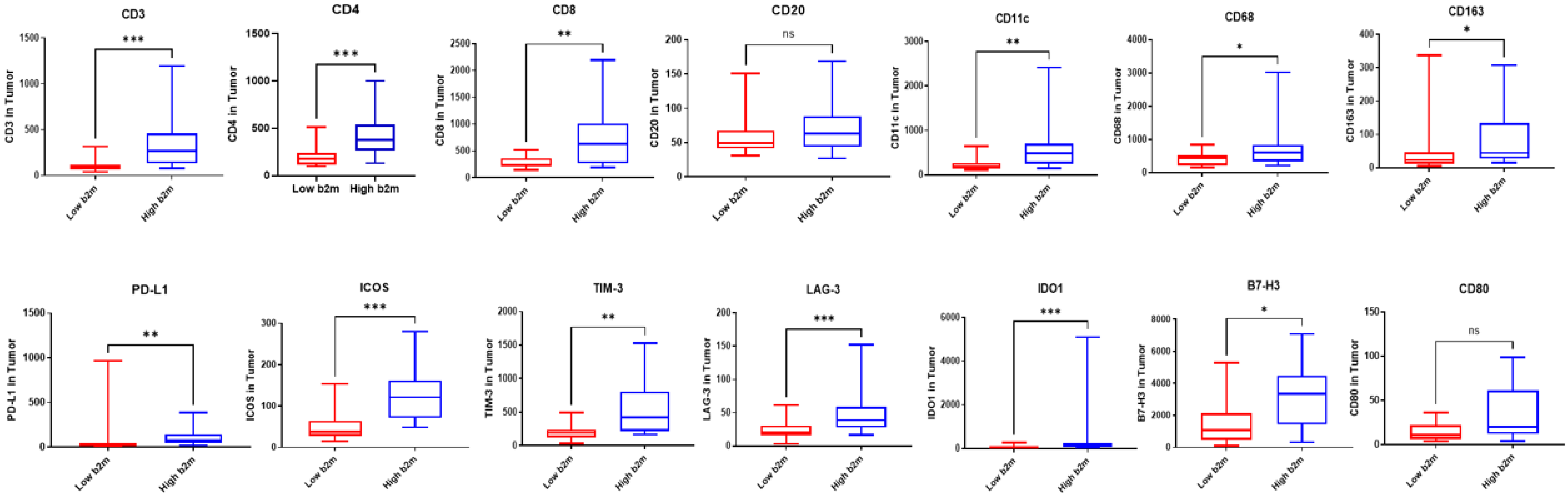

Supplemental Figure 6. Differential expression of immune-cell markers and immune-checkpoint molecules between “B2M-high” and “B2M-low” tumors, in the tumor compartment. (unadjusted P values; * $P < 0.05$, ** $P < 0.01$, *** $P < 0.001$), (B2M, beta-2 microglobulin)
