# Supplementary material for: Digital Spatial Profiling Links Beta-2-microglobulin Expression with Immune Checkpoint Blockade Outcomes in Head and Neck Squamous Cell Carcinoma
Source: Cancer Res Commun. 2023 Apr 11;3(4):558–63. doi: 10.1158/2767-9764.CRC-22-0299 (PMC10088911; doi:10.1158/2767-9764.CRC-22-0299)
Supplement: Supplemental Table 1 — clinical characteristics of discovery (Athens) and validation (Yale) cohorts [file crc-22-0299-s07.pdf]

	Discovery (Athens) cohort, N (%)	Validation (Yale) cohort, N (%)
**Age (y)**		
Median [Min, Max]	63.3 [34.6, 89.3]	60.1 [41.5, 82.3]
**Sex**		
Male	41 (82.0%)	26 (89.7%)
Female	9 (18.0%)	3 (10.3%)
**Smoking (current/former)**		
Yes	42 (84.0%)	21 (72.4%)
No	8 (16.0%)	8 (27.6%)
**Alcohol (current/former)**		
Yes	18 (36.0%)	12 (41.4%)
No	32 (64.0%)	17 (58.6%)
**Primary Site**		
Oral Cavity	23 (46.0%)	6 (20.7%)
Larynx	15 (30.0%)	6 (20.7%)
Oropharynx	9 (18.0%)	15 (51.7%)
Other	3 (6.0%	2 (6.9%)
**Immunotherapy agent**		
Nivolumab	45 (90.0%)	10 (34.5%)
Pembrolizumab	1 (2.0%)	19 (65.5%)
Other	4 (8.0%)	na
**Immunotherapy line of treatment**		
First	22 (44.0%)	21 (72.4%)
Second	28 (56.0%)	7 (24.1%)
Third	na	1 (3.4%)
**Best overall response**		
Complete response	3 (6.0%)	3 (10.3%)
Partial response	7 (14.0%)	7 (24.1%)
Stable disease	8 (16.0%)	5 (17.2%)
Progressive disease	32 (64.0%)	10 (34.5%)
Non evaluable	na	4 (13.8%)
**Progression free survival (months)**		
Mean (SD)	6.85 (9.25)	9.28 (10.8)
**Overall survival (months)**		
Mean (SD)	10.8 (11.2)	13.5 (11.4)

Supplemental Table 1. Clinical characteristics of the discovery (Athens, YTMA496) and validation (Yale, YTMA523) cohorts.
