# Supplementary material for: Digital Spatial Profiling Links Beta-2-microglobulin Expression with Immune Checkpoint Blockade Outcomes in Head and Neck Squamous Cell Carcinoma
Source: Cancer Res Commun. 2023 Apr 11;3(4):558–63. doi: 10.1158/2767-9764.CRC-22-0299 (PMC10088911; doi:10.1158/2767-9764.CRC-22-0299)
Supplement: Supplemental Table 2 — Target-protein panels included in the GeoMxTM Immuno-Oncology Human Protein Assay [file crc-22-0299-s08.pdf]

Human protein panels	Immune cell profiling	ITx Drug Target	Immune Activation Status	Cell Death	Immune Cell typing	Pan-Tumor	MAPK Signaling	Control proteins
Markers	Beta-2-microglobulin<br>CD11c<br>CD20<br>CD3<br>CD4<br>CD45<br>CD56<br>CD68<br>CD8<br>CTLA4<br>Granzyme B<br>Ki-67<br>PD-1<br>PD-L1<br>Pan-cytokeratin<br>HLA-DR<br>SMA<br>Fibronectin	4-1BB<br>ARG1<br>B7-H3<br>GITR<br>IDO1<br>LAG3<br>OX40L<br>STING<br>TIM-3<br>VISTA	CD127<br>CD25<br>CD27<br>CD40<br>CD44<br>CD80<br>ICOS<br>PD-L2	BAD<br>BCL6<br>BCLXL<br>BIM<br>CD95/Fas<br>GZMA<br>P53<br>PARP<br>Cleaved Caspase 9	CD14<br>CD163<br>CD34<br>CD45RO<br>CD66b<br>FAP alpha<br>FOXP3	BCL-2<br>EpCAM<br>ER alpha<br>HER2/ERBB2<br>MART1<br>NY-ESO-1<br>PR<br>PTEN<br>S100B	EGFR<br>Pan-RAS<br>BRAF<br>Phospho-c-RAF (S338)<br>Phospho-JNK (T183/Y185)<br>Phospho-MEK1 (S217/S221)<br>Phospho-p38 MAPK (T180/Y182)<br>Phospho-p44/42 MAPK ERK1/2 (T202/Y204)<br>P44/42 MAPK ERK1/2<br>Phospho-p90 RSK (T359/S363)	Histone H3<br>S6<br>GAPDH<br>Ms IgG1<br>Ms IgG2a<br>Rb IgG

Supplemental Table 2. Target-protein panels included in the GeoMx™ Immuno-Oncology Human Protein Assay. ITx, immunotherapy.
